# The Therapeutic Prospects of Targeting IL-1R1 for the Modulation of Neuroinflammation in Central Nervous System Disorders

**DOI:** 10.3390/ijms23031731

**Published:** 2022-02-02

**Authors:** João P. Luís, Carlos J. V. Simões, Rui M. M. Brito

**Affiliations:** 1 Coimbra Chemistry Center-Institute of Molecular Sciences (CQC-IMS), Department of Chemistry, University of Coimbra, 3004-535 Coimbra, Portugal; jluis@student.ff.uc.pt (J.P.L.); csimoes@qui.uc.pt (C.J.V.S.); 2 BSIM Therapeutics, Instituto Pedro Nunes, 3030-199 Coimbra, Portugal

**Keywords:** interleukin-1, interleukin-1 receptor type 1, neuroinflammation, CNS diseases, therapeutic target

## Abstract

The interleukin-1 receptor type 1 (IL-1R1) holds pivotal roles in the immune system, as it is positioned at the “epicenter” of the inflammatory signaling networks. Increased levels of the cytokine IL-1 are a recognized feature of the immune response in the central nervous system (CNS) during injury and disease, i.e., neuroinflammation. Despite IL-1/IL-1R1 signaling within the CNS having been the subject of several studies, the roles of IL-1R1 in the CNS cellular milieu still cause controversy. Without much doubt, however, the persistent activation of the IL-1/IL-1R1 signaling pathway is intimately linked with the pathogenesis of a plethora of CNS disease states, ranging from Alzheimer’s disease (AD), Parkinson’s disease (PD), amyotrophic lateral sclerosis (ALS) and multiple sclerosis (MS), all the way to schizophrenia and prion diseases. Importantly, a growing body of evidence is showing that blocking IL-1R1 signaling via pharmacological or genetic means in different experimental models of said CNS diseases leads to reduced neuroinflammation and delayed disease progression. The aim of this paper is to review the recent progress in the study of the biological roles of IL-1R1, as well as to highlight key aspects that render IL-1R1 a promising target for the development of novel disease-modifying treatments for multiple CNS indications.

## 1. Introduction

One of the most well-established groups of cytokines capable of orchestrating inflammatory responses by inducing the expression of pro-inflammatory molecules in both peripheral (PNS) and central nervous system (CNS) environments is the interleukin-1 (IL-1) family. Within this group, the first interleukin ever purified, IL-1, is found in two distinct isoforms: IL-1α and IL-1β [1,2]. It stands as a pleiotropic cytokine with multiple biological activities, far from restricted to promoting inflammation, including (i) the development and maturation of immune cells, (ii) fever, (iii) the regulation of insulin levels and lipid metabolism and (iv) the regulation of stress response through the modulation of the hypothalamic–pituitary–adrenal (HPA) axis [3,4]. The biological effects of IL-1α and IL-1β are mediated by binding to the IL-1 receptor type 1 (IL-1R1). IL-1R1 is a membrane-bound protein that may also be cleaved by matrix metalloproteases to a soluble, circulating form. Both the membrane-bound and the soluble forms of IL-1R1 are biologically active, regulating the inflammatory response through agonistic and antagonistic modulation of cytokine activity [5,6]. Importantly, dysregulation of IL-1 activity may result in exacerbated inflammation and increased tissue damage [7,8,9]. The IL-1 inflammatory cytokine has been shown to be upregulated in a wide range of human pathologies, ranging from monogenic autoinflammatory diseases such as cryopyrin-associated periodic syndrome (CAPS) and familial Mediterranean fever (FMF), to rheumatoid arthritis, type 2 diabetes mellitus, cancer and neurodegenerative diseases [8,10,11]. Hence, the regulatory role of IL-1R1 in the inflammatory cascades has attracted a lot of interest and attention in the drug discovery field since its discovery.

The increasing awareness of the involvement of the immune system, not only in the progression but also directly related to the onset of CNS diseases, has fueled a renewed interest in the field in recent years [12,13,14]. Indeed, neuroinflammation has been linked to Parkinson’s disease (PD) [15], Alzheimer’s disease (AD) [16,17], amyotrophic lateral sclerosis (ALS) [18] and other CNS disorders, such as multiple sclerosis [19], traumatic brain injury [20], depression [21] and schizophrenia [22], among others. The involvement of the neuroinflammatory landscape in such diseases reinforces the notion that overstimulation of the immune response is a major determinant in the pathophysiology of these diseases. Three main factors appear to be key across all the cited diseases, which in many respects are complementary in nature: (i) cytokines, whether in a damaging or protective capacity (or both), are involved in the different disease stages, holding pleiotropic and systemic effects and displaying variations in number and phenotypic features in all cell types; (ii) glial cell activation is an early and persistent feature in the course of the disease, whose phenotype and function may change over time; (iii) a whole constellation of neuron–glia interactions sustain and propagate the neuroinflammatory response [13]. While current treatments for most CNS pathologies arguably have limited efficacy, IL-1R1 represents a worthwhile target in the search for neuroinflammation-associated CNS diseases therapeutics, due to its central role in the immune response.

In this article, we review what we see as major advances in the understanding of the roles of IL-1R1 in the CNS, the cellular signaling mechanisms of IL-1R1 and current pharmacotherapy, as well as recent evidence strongly suggesting that IL-1R1 may be an important modulator in the CNS under pathophysiological conditions.

## 2. IL-1R1 Signaling

Human IL-1R1 is encoded by the *IL-1R1* gene, which is located on the long arm of chromosome 2 at band 2q12. IL-1R1 is synthesized as a biologically active 80 kDa transmembrane protein and belongs to the interleukin-1 receptor (IL-1R) family, whose structural hallmark feature consists of the presence of immunoglobulin (Ig)-like domains in the extracellular ligand-binding region of the receptors. IL-1R1 also contains a transmembrane α-helix and a cytoplasmic TIR domain responsible for initiating intracellular signaling [23]. In addition, a second IL-1 receptor is known. IL-1 receptor type 2 (IL-1R2) is a 66 kDa glycoprotein characterized by the lack of an intracellular TIR domain, functioning as a decoy receptor for IL-1 [24,25]. Murine and human IL-1R1 proteins show 69% identity at the amino acid level. In both species, the *IL-1R1* and *IL-1R2* genes are adjacent, encoding similar transmembrane regions but only having 28% homology in their extracellular domains [26].

**Figure 1:**
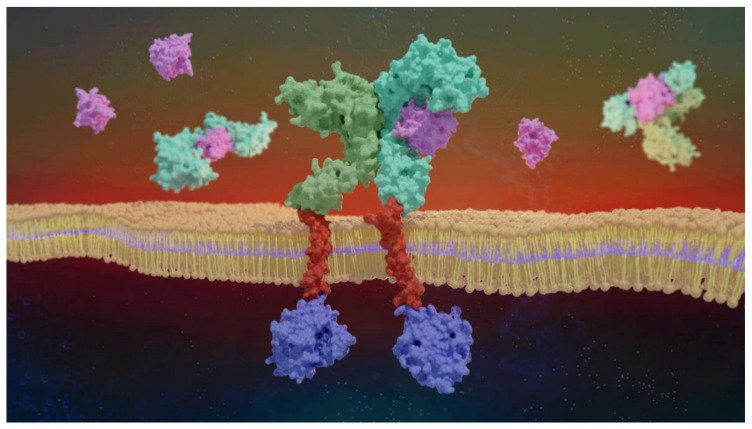
An illustration of the IL-1β/IL-1R1/IL-1RAcP ternary complex. Binding of the IL-1 cytokine (colored in pink) to the membrane-bound IL-1R1-ECD (colored in green) recruits transmembrane IL-1RAcP (colored in lime), initiating intracellular signaling via the TIR domains (colored in blue). Both IL-1R1 and IL-1RAcP can participate in the negative regulation of IL-1 signaling when cleaved to their soluble ECD forms. The lipid bilayer (cell membrane) is represented in yellow and transmembrane domains are colored in red. The image was generated using imported VMD visualization states [33] uploaded in the Blender software [34]. Figure abbreviations: IL-1, interleukin-1; IL-1R1, interleukin-1 receptor type 1; IL-1R1-ECD, extracellular domain of IL-1R1; IL-1RAcp, interleukin-1 receptor accessory protein; TIR domain, the toll-interleukin-1 receptor homology domain.

**Figure 2:**
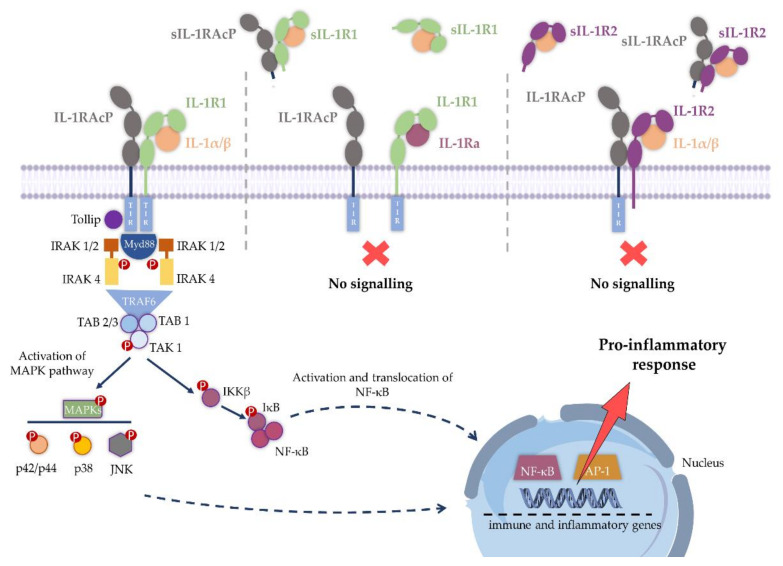
Schematic representation of IL-1 signaling. Upon binding of IL-1α/β to the extracellular domain of membrane-bound receptor IL-1R1, IL-1RAcP is recruited and signaling is initiated by the interaction of the intracellular TIR domains of the two polypeptide chains. When IL-1Ra binds IL-1R1, the IL-1RAcP is not recruited, thereby blocking signaling. Similarly, when IL-1β binds IL-1R2, no signaling occurs as IL-1R2 lacks a cytoplasmic TIR domain. IL-1 signaling may also be inhibited by soluble forms of the receptor, sIL-1R1 and sIL-1R2, lacking the transmembrane and intracellular regions of the native form, binding both IL-1α, IL-1β and sIL-1RAcP. Adapted from [39]. Figure abbreviations: IL-1, interleukin-1; IL-1R1, interleukin-1 receptor type 1; IL-1Ra, interleukin 1 receptor antagonist; IL-1RAcP, interleukin-1 receptor accessory protein; IL-1R2, interleukin-1 receptor type 2; TIR domain, the toll-interleukin-1 receptor homology domain; IRAK, interleukin-1 receptor-associated kinases; TRAF6, tumor necrosis factor receptor associated factor 6; TAK1, transforming growth factor-β-activated kinase 1; TAB, TAK1-binding proteins; MAPK, mitogen-activated protein kinases; p42/p44 MAPK, p42/p44 mitogen-activated protein kinases; p38 MAPK, p38 mitogen-activated protein kinases; JNK., c-Jun N-terminal kinase; IKKβ, I kappa B kinase β; NF-κB, nuclear factor-kappa B; AP-1, activator protein 1.

Within the complex regulatory networks of IL-1 pathways, the soluble cytokines IL-1α and IL-1β interact with the extracellular domain of IL-1R1, triggering the recruitment of an accessory receptor, the IL-1RAcP, resulting in a functional receptor complex that initiates IL-1R1 signaling cascades (Figure 1) [27,28]. The heterotrimeric IL-1/IL-1R1/IL-1RAcP complex leads to the dimerization of the TIR domains of IL-1R1 and IL-1RAcP proteins, providing an anchor point for the recruitment of the myeloid differentiation primary response protein 88 (Myd88) (Figure 2). This protein–protein interaction sparks the recruitment of other signaling molecules such as the IL-1R-associated kinases (IRAKs) and TNF-receptor-associated factor 6 (TRAF6) to the protein complex. Subsequently, multiple intracellular phosphorylation and ubiquitination processes culminate in the activation of mitogen-activated protein kinase (MAPK) p38, the c-Jun N-terminal kinase (JNK) and nuclear factor kappa B (NF-κB). These changes result in the upregulation of mRNA transcription for inflammation-associated genes encoding IL-6, IL-8, inducible nitric oxide synthase (iNOS), monocyte chemoattractant protein-1 (MCP-1), cyclooxygenase-2 (COX-2), IκBα, IL-1α, IL-1β and MAPK phosphatase 1 (MKP-1) [3,29,30]. Besides agonists IL-1α and IL-1β, IL-1R1 also binds an endogenous antagonist, IL-1Ra, which is not able to trigger IL-1R1 association with IL-1RAcP, thereby competitively blocking IL-1 signaling through IL-1R1 binding. IL-1R1 binds the three ligands, IL-1α, IL-1β and IL-1Ra, with comparable affinities (0.1 to 1 nM K_d_) [6,31,32].

The biological activities of IL-1 are also modulated through soluble IL-1 receptors (sIL-1R) [35,36]. Post-translational shedding of the extracellular Ig domains is the major mechanism responsible for the release of soluble IL-1 receptors (sIL-1R1, sIL-R2 and sIL-1RAcP) from cell membranes [5,37]. In the extracellular environment, the soluble extracellular IL-1R1 domain (sIL-1R1) can capture IL-1 molecules in solution, acting as a decoy, preventing signal transduction. Furthermore, sIL-1R1 binds IL-1Ra, limiting the effects of this IL-1 receptor antagonist on the membrane-bound receptor. Similarly, sIL-1R2 binds IL-1β with high affinity and recruits sIL-1RAcP, but does not initiate intracellular signaling, also acting as a decoy receptor. In contrast to sIL-1R1, IL-1Ra binds with weak affinity to this soluble decoy and both IL-1Ra and sIL-1R2 cooperate in the negative regulation of IL-1 [25,38].

The amplifying power of this signaling pathway is remarkable. The interaction of IL-1 with transmembrane IL-1R1 catalyze the activity of numerous intracellular molecules with fundamental mechanisms of immunoregulation. The domino-like cascade of IL-1 activity and signal amplification continues to intensify at every step of the pathway. Importantly, positive modulators (receptor agonists) and negative modulators (receptor antagonists, decoy receptors) cooperate to regulate the inflammatory response. However, an uncontrolled and sustained activation of immune cells by abnormal protein aggregates, such as amyloid-β-peptide (Aβ) plaques and α-synuclein aggregates, or by lipopolysaccharide (LPS), or even injury, can drive IL-1R1 signaling dysregulation, generating a strong pro-inflammatory microenvironment, thus resulting in pathological outcomes. IL-1 signaling has been implicated in several inflammatory diseases, prompting the need to find druggable therapeutic targets within the components of the IL-1 inflammatory pathway [4,36].

## 3. Expression of IL-1R1 in the CNS

Produced mainly by cells of the immune system, IL-1R1 operates in various parts of the body as a key cytokine receptor in inflammation and immune responses. The involvement of IL-1R1 in the brain has been linked to the pyramidal cell layer of the hippocampus, dentate gyrus, cerebellum, pituitary gland and hypothalamus [40,41,42]. Within the CNS cellular machinery, functional IL-1R1 is highly expressed among vascular endothelial cells, with lower but detectable expression in microglia, astrocytes and neurons [43,44]. In a recent study, Liu et al. employed genetic knock-in reporter mice to investigate the IL-1R1 cytoarchitecture and cell-type-specific roles in the CNS [45]. They demonstrated that ventricular IL-1R1 regulates monocyte recruitment, endothelial and ventricular IL-1R1 regulates IL-1-induced microglial activation and endothelial IL-1R1 mediates sickness behavior, leukocyte recruitment and neurogenesis [45]. Small amounts of mRNA have been found in glial cells under basal conditions [46], contradicting other studies that showed that IL-1R1-mediated signaling drives the activation of astrocytes [47]. However, the presence of IL-1R1 in microglia is not entirely consensual. Indeed, the expression of this receptor was reported in some in vitro studies [46,48] but was not confirmed in others [45,49]. Nevertheless, microglial IL-1R1 expression has been reported to be increased in different in vivo models of neuroinflammation, providing evidence of IL-1R1’s important role in the molecular mechanisms of the neuroinflammatory response [50,51,52,53,54]. Importantly, Basu et al. demonstrated in IL-1R1 null mice that this receptor is fundamental for the activation of microglia and the induction of pro-inflammatory mediators, such as IL-6, in response to brain injury [55]. These observations indicated that IL-1R1 production in the CNS is more strongly associated with an activated status of human glial cells. As such, under pathological conditions, IL-1R1 may be induced by IL-1β itself, elevating the mRNA levels of this receptor on glial cells.

**Figure 3:**
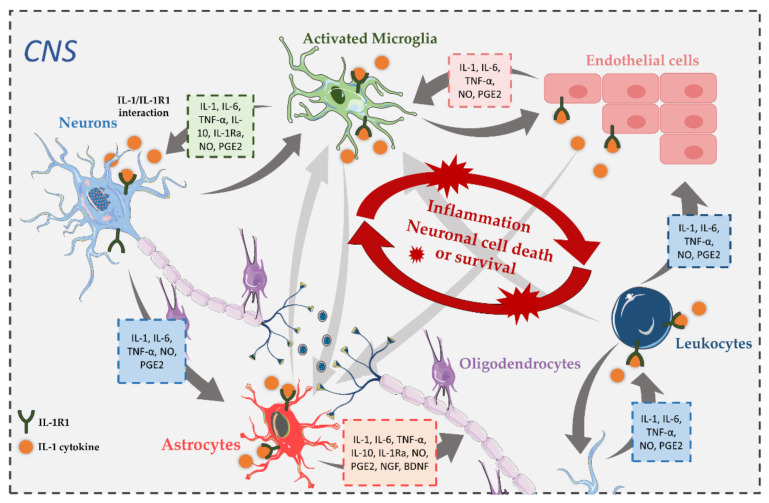
CNS cellular communication of IL-1 signaling in neuronal injury. In the normal brain, the expression of IL-1 is low, although acute CNS injury causes a sharp increase in IL-1 production. Different CNS cell types express IL-1R1, thereby enabling them to respond to IL-1β in an autocrine manner, as well as in a paracrine manner, after neuronal injury. Upon IL-1/IL-1R1 interactions, these different cells can produce a wide range of pro-inflammatory and immune-regulatory mediators that contribute to neuronal death or survival through different signal transduction pathways. The figure was prepared using Servier Medical Art. Figure abbreviations: CNS, central nervous system; IL-1, interleukin-1; IL-6, interleukin-6; IL-10, interleukin-10; IL-1Ra, interleukin-1 receptor antagonist; IL-1R1, interleukin-1 receptor type 1; TNF-α, tumor necrosis factor-α; NO, nitric oxide; PGE2, prostaglandin E2; NGF, nerve growth factor; BDNF, brain-derived neurotrophic factor.

Once IL-1 is synthetized in the brain, it is plausible to deem a function for it in the biology of the CNS. Indeed, IL-1 has been associated with the regulation of fever [56], sleep [57], neurogenesis [58] and the modulation of long-term potentiation [59]. Interestingly, low levels of IL-1 have been hypothesized to help consolidate memory, whereas higher levels of IL-1 impair sensory function and memory [60,61]. In a neuroinflammatory setting, IL-1, in particular IL-1β, is intimately involved in the CNS’s innate immune response to injury, infections or exposure to misfolded proteins. Once activated in response to injury, microglia and astrocytes represent the main source of IL-1β, and when secreted it can further stimulate its own production in an autocrine or paracrine fashion, along with the release of other pro-inflammatory mediators, by binding to IL-1R1 [62]. Importantly, this dynamic signaling network of IL-1β production in activated glia ensures that injury signals are further propagated in the cellular milieu, driving potent neuroinflammatory changes in the brain (Figure 3).

## 4. The Role and Involvement of IL-1 Pathways in CNS Disorders

Excessive IL-1R1 activation underlies a wide range of different CNS pathological conditions characterized by strong neuroinflammatory components such as AD [63,64,65], PD [66,67], ALS [68,69], multiple sclerosis [70,71,72], traumatic brain injury [54,73], Creutzfeldt-Jakob disease [74], HIV-1 encephalitis [75] and age-related macular degeneration [76]. The biochemical basis by which IL-1 exerts neurodegeneration effects is still elusive; however, some clues to the possible mechanism(s) are emerging [7,42,77]. In this section, we describe evidence of the involvement of IL-1R1 in CNS diseases.

### 4.1. IL-1R1 in Alzheimer’s Disease

One of the most well-known examples of a disease featuring neuroinflammation is AD, a progressive, neurodegenerative, age-associated condition accounting for 60–70% of the estimated 50 million people globally who suffer from dementia [78]. The pathogenesis of AD requires the contribution of multiple factors, of which two have been considered the main hallmarks: the presence of Aβ plaques and neurofibrillary tangles (aggregates of hyperphosphorylated tau protein) in the brain. Among the many inflammatory pathways implicated in the disease, signaling through IL-1β reportedly exacerbates AD pathogenesis [79,80]. At the molecular level, IL-1β is regulated by the NLR family pyrin domain containing 3 (NLRP3) inflammasome, a central hub for cytokine production that initiates downstream inflammatory cascades in response to endogenous danger signals. Upon stimulation, this inflammasome assembles into a multiprotein complex, activating the proteolytic processing of precursor proteins, leading to the biological active forms of IL-1β and IL-18, two important pro-inflammatory mediators that are significantly increased in AD brains [65,81,82,83]. In a recent study, Ising and colleagues observed that mice lacking the NLRP3 inflammasome fail to induce the calcium–calmodulin-dependent protein kinase type II subunit alpha (CaMKIIα), which plays a critical role in tau hyperphosphorylation and aggregation in AD. In contrast, microglia-derived IL-1β via microglial NLRP3 activation increases the levels of CaMKIIα and promotes tau aggregation in neurons. Importantly, blocking the IL-1β signaling pathway through the administration of IL-1Ra, the natural antagonist that directly targets IL-1R1, hampered the effects of CaMKIIα, and consequently tau phosphorylation [84]. These data elucidate the role of microglial IL-1β in tau pathology and are consistent with results from other studies [85,86].

In parallel, another recent study established a link between the levels of IL-1R1 and the endosomal adaptor target of Myb1 (TOM1) in the brains of AD patients. TOM1 is a cytosolic protein that internalizes the IL-1R1–IL-1β complex into endosomes, ensuring the downregulation of inflammatory responses and avoiding excessive IL-1R1 signaling [87]. Martini et al. found that TOM1 is significantly reduced in human AD brains and is associated with a corresponding increase in the levels of IL-1R1. The levels of this receptor were significantly increased in primary hippocampal neuronal cultures after treatment with Aβ-derived diffusible ligands. Likewise, in AD transgenic mouse brains, decreased levels of TOM1 were also accompanied by increased IL-1R1 and IL-1β levels. Consistent with these results, AD transgenic mice injected with adeno-associated virus (AAV) constructs designed to overexpress or knock-down TOM1 showed in the latter a shift towards a more pro-inflammatory immune microenvironment, increased Aβ plaque deposition, impaired microglia phagocytosis and increased IL-1R1 expression on neuronal cell membranes. Interestingly, no significant changes in other inflammatory receptors, such as toll-like receptor 4 (TLR4) and tumor necrosis factor receptor (TNFR), were observed, suggesting that TOM1 is intimately linked to IL-1R1 in AD pathophysiology. On the contrary, overexpressing TOM1 reduced neuronal IL-1R1, improved microglial phagocytic efficiency and reduced Aβ burden [88]. Together, the authors point to an important mechanism through which IL-1β and IL-1R1 are related to Aβ accumulation, suggesting that therapeutics stimulating the TOM1 signaling pathway could be an appealing strategy to counteract the persistent activation of the IL-1/IL-1R1 complex, contributing to a homeostatic immune response in AD.

Italiani et al. measured the levels of the inflammation-related cytokines and receptors of the IL-1 family in serum samples of subjects with AD [65]. The soluble IL-1R1 circulating form, sIL-1R1, together with the pro-inflammatory cytokines IL-1α and IL-1β, the antagonist IL-1Ra and the accessory receptor sIL-1RAcP, were shown to be elevated in the serum samples of subjects with AD, contrasting with the levels verified in patients with subjective memory complaints (SMC) and mild cognitive impairment (MCI). In this study, the researchers suggested that the underlying inflammatory reaction linked with AD has selectively activated the IL-1R1-cleaving protease, leading to increased levels of sIL-1R1. However, since sIL-1R1 binds IL-1Ra with high affinity, decreasing its capacity to capture IL-1 cytokines in solution, it may counteract the regulatory mechanisms to control IL-1/IL-1R1-induced inflammation, and consequently to enhance neuroinflammatory responses in AD. [65].

### 4.2. IL-1R1 in Parkinson’s Disease

The core of PD is characteristically ascribed to the accumulation of aggregated forms of misfolded α-synuclein (Lewy bodies) in the *substantia nigra* (SN) area of the brain. These abnormal protein aggregates have been deemed a critical factor responsible for cellular homeostasis imbalance and progressive degeneration of dopaminergic neurons. However, although these molecular mechanisms underlying α-synuclein propagation in PD still remain elusive, the involvement of numerous innate and adaptive inflammatory processes in the brain and in the periphery of PD patients is increasingly recognized [15,89]. Increased IL-1β expression has been found in the cerebrospinal fluid (CSF) [66], striatum [90] and SN of PD patients [91]. Mice studies based on the injection of LPS or the adenovirus expressing IL-1 into the SN, which had been previously exposed to 6-hydroxydopamine (6-OHDA)—a neurotoxin that is commonly used to induce PD—led to the activation of microglia towards a pro-inflammatory phenotype, increasing IL-1β secretion and the chemokine MCP-1 and decreasing the anti-inflammatory cytokine levels of IL-4. Notably, IL-1Ra administration reduced LPS-induced tumor necrosis factor-α (TNF-α) and interferon-γ (IFN-γ) release and counteracted the contribution of 6-OHDA to the excessive loss of dopaminergic neurons [92,93,94]. More recently, Stojakovic et al. employed *IL-1Ra* knockout mice to evaluate the role of the IL-1 pathway in dopaminergic neurodegeneration and motor skills during aging [95]. In mice lacking IL-1Ra, the over-activation of the IL-1 pathway was associated with sustained activation of microglia and exacerbated neuroinflammation, resulting in dopamine loss and parkinsonism during aging [95]. According to the authors, therapeutic strategies modulating IL-1 activity may have neuroprotective effects in PD patients.

A recent work suggests that IL-1β/IL-1R1 signaling in the olfactory bulb is associated with a higher degree of α-synucleinopathy, which spreads to the SN, triggering the loss of dopaminergic neurons. Using animal models, Niu et al. verified that LPS intranasal infusion sparked the activation of microglia in the olfactory bulb, SN and striatum in an IL-1R1-dependent manner, increasing the levels of phosphorylated and total α-synuclein in the olfactory bulb and SN of mice [96]. Furthermore, mice showed a reduced number of tyrosine hydroxylase (TH)-immunoreactive cells, which produce either adrenaline–noradrenaline or dopamine, indicating a concomitant loss of dopaminergic neurons and motor dysfunction. Interestingly, LPS treatment in mice lacking IL-1R1 did not increase the expression of α-synuclein or the downstream consequences, highlighting the key role of IL-1R1 in the propagation of α-synuclein pathology from the olfactory bulb to the SN, supporting the possible involvement of this pro-inflammatory receptor in PD progression [96].

### 4.3. IL-1R1 in Amyotrophic Lateral Sclerosis

The tangled network of cytokines and immune molecules, glial activation and sustained neuroinflammation emerge again as prominent features in ALS. This non-cell-autonomous disease is characterized by motor neuron degeneration leading to progressive muscle weakness, paralysis and ultimately death [18,97]. Mutations in approximately a dozen genes have already been identified as risk factors for ALS in humans. Of these, the main ones are Cu, Zn superoxide dismutase 1 (*SOD1*), TAR DNA binding protein 43 (*TARDBP*), fused in sarcoma (*FUS*) and chromosome 9 open reading frame 72 (*C9orf72*), responsible for 5–10% of ALS cases [18,98]. The remaining 90–95% of ALS are sporadic in nature, meaning there is no known cause or mechanism for the disease. Amongst the several intrinsic mechanisms proposed for neuronal death in ALS, the involvement of inflammation is now believed to be essential to disease progression [99,100]. Elevated concentrations of IL-1β have been reported in glial cells expressing G93A mutant SOD1 [101,102] and in G93A-SOD1 transgenic mice [69,103,104,105]. Meissner et al. reported that mutant SOD1 can directly activate microglial cells to enhance caspase-1 levels and the secretion of mature IL-1β after cytoplasmic accumulation, inducing inflammation [69]. By crossing G93A-SOD1 transgenic mice with caspase-1- or IL-1β-deficient mice, they verified an extended lifespan of mutant SOD1 transgenic animals, as well as reduced microgliosis and astrogliosis coinciding with an increased number of motor neurons. Moreover, treatment of transgenic mice with the recombinant IL-1R1 antagonist anakinra resulted in a similar prolonged survival and slowed disease progression, as seen in IL-1β-deficient mice [68,69].

Even though antagonizing IL-1 showed promising results in animal models of ALS, human data are somehow inconsistent. For instance, a study evaluating inflammation-related IL-1 cytokine levels in sporadic ALS patients found that only IL-18 and not IL-1β levels were increased in the serum samples of these patients [106]. Furthermore, a preclinical trial involving 17 ALS patients to assess the safety profile and immunomodulatory effects of anakinra showed no significant improvement in disease progression, although the recombinant biologic was deemed safe in these patients. Although lower levels of cytokines and the inflammatory marker fibrinogen were achieved during the first 6 months of treatment, these inflammatory mediators returned to higher levels in the second part of the study [107]. On the other hand, a recent study evaluating the peripheral and central immune cell profiles in ALS patients found that pro-inflammatory serum cytokines IL-1β, IL-6 and IFN-γ were increased while the anti-inflammatory cytokine IL-10 was decreased, highlighting a shift toward a pro-inflammatory microenvironment [108]. Recently, Yuan et al. established a potential causal inverse association between circulating IL-1ra levels and ALS risk, suggesting that IL-1 inhibitors may lower the risk of ALS [109]. These contradictory studies corroborate the fact that the influence of the IL-1/IL-1R1 signaling pathway is still poorly understood within the neuroinflammation landscape underlying ALS, emphasizing the need for large prospective studies to confirm such findings.

### 4.4. IL-1R1 in Multiple Sclerosis

Multiple sclerosis (MS) is as a chronic autoimmune disease characterized by inflammation and demyelination of the CNS. Amongst the inflammatory signaling pathways involved in this disease, the IL-1/IL-1R1 signaling network is believed to be essential for its pathophysiology [110]. In particular, elevated levels of IL-1β were detected in chronic active lesions and CSF samples of MS patients [70,71,72,111,112] and in animal models of experimental autoimmune encephalomyelitis (EAE)—an autoimmune model particularly used to study myelin demyelination, neuroinflammation and immune system activation [113,114]. The biochemical basis by which IL-1 exerts pathogenic effects is still elusive, although IL-1R1 signaling has been linked to the differentiation of T helper (Th) 17 cells, which are essential for the development of autoimmune disease [115,116,117]. Indeed, IL-1R1 signaling promotes the pathogenicity of autoimmune Th17 cells by synergizing with IL-6 and IL-23 [118]. The expression of IL-1R1 was found to be elevated in Th cells derived from MS patients in comparison to those from healthy donors [119,120]. Interestingly, IL-1β treatment in Th17 cells is reported to upregulate the production of the granulocyte macrophage colony-stimulating factor (GM-CSF), an important cytokine that induces the activation and migration of myeloid cells to inflammation sites [121]. EAE incidence and the onset of clinical signs were reduced in *IL-1R1* knockout mice, suggesting that IL-1R1 expression in T cells is required for the Th17 cell differentiation [116,122,123]. These observations are in agreement with other reports employing treatment with IL-1Ra or the administration of sIL-1R1 in EAE, revealing a significant delay of EAE clinical signs, reduced extent of paralysis and reduced evidence of lesions in the CNS [124,125,126,127].

There is evidence that endothelial IL-1R1 also plays a preponderant role in EAE. Endothelial cells (ECs) of the BBB cellular machinery are known to express high levels of IL-1R1, specifically on the pial venous plexus, which corresponds to the primary site of myeloid cell infiltration during acute EAE [127,128]. In fact, such cells transmigrate across the BBB, secreting IL-1, which triggers endothelial IL-1R1 signaling and consequently induces the recruitment of neutrophils and T cells into the CNS during neuroinflammation [129,130]. Li et al. demonstrated that knockdown of *IL-1R1* in ECs of mice results in decreased incidence, severity and a delayed onset of EAE [131]. Accordingly, a recent study seeking to understand the cell-type-specific role of IL-1 signaling in EAE pathogenesis showed that the deletion of IL-1R1 in ECs of transgenic mice reduced EAE severity, whereas IL-1 signaling in astrocytes or microglia was redundant for EAE development, emphasizing the importance of endothelial IL-1R1 in CNS autoimmunity [132]. Paré et al. presented an in vitro transmigration assay based on human brain microvascular endothelial cells, showing that deletion of the *IL-1R1* gene or treatment with anakinra or an anti–IL-1β blocking antibody reduced the transmigration of monocytes and neutrophils across the monolayer [130]. Their studies suggest that the IL-1β/IL-1R1 signaling is vital for the recruitment of myeloid cells across the CNS [130]. Whether endothelial IL-1R1 is a critical mediator of MS pathogenesis is still a matter of debate, but these studies show that IL-1R1 activation in endothelial cells is fundamental for myeloid cell recruitment and BBB disruption, amplifying the neuroinflammatory response. Together, growing evidence from EAE and MS sustain the fact that targeting endothelial IL-1R1 may open therapeutic avenues for neuroprotection in MS.

### 4.5. IL-1R1 in Schizophrenia

Schizophrenia is a complex neuropsychiatric disorder with a heterogeneous etiology involving the contributions of multiple genetic and environmental risk factors. While the mechanisms involved in the pathogenesis of this disease are still unclear, the influence of immunological processes resulting in inflammation has been increasingly recognized [133,134]. Several studies have evaluated the levels of the IL-1 cytokines in schizophrenia, reporting increased levels of IL-1β [135,136,137,138] and IL-1Ra [139,140,141] in the plasma of schizophrenia patients, as well as decreased levels of IL-1α [142]. To date very little is known about IL-1R1 in schizophrenia. Pandey et al. found that the mRNA levels of *IL-1R1*, *IL-6*, *TNF-α*, *TNFR1* and *TNFR2* were significantly increased in lymphocytes of schizophrenia patients compared with control subjects, suggesting that these pro-inflammatory players may be useful biomarkers or targets for therapeutic approaches [143]. Furthermore, in the CSF of patients with first-episode schizophrenia, a marked elevation of IL-1β was detected, with authors suggesting that increased levels of IL-1β, and consequently IL-1β/IL-1R1 signaling-mediated effects, may be normalized or downregulated during the disease progression or during prolonged antipsychotic treatment [144]. Contrary to many reports pointing to upregulated levels of IL-1β or IL-1Ra in schizophrenia, other studies found decreased or no significant alterations in the levels of these cytokines [140,145,146]. As such, the results and observations concerning IL-1 cytokines and their receptors are still vastly inconclusive and must be interpreted with caution [147].

### 4.6. IL-1R1 in Epilepsy

Several lines of evidence have shown that IL-1β exacerbates seizure activity in experimental models of epilepsy [148,149,150,151], leading to the assumption that there is an involvement of IL-1 signaling in the pathophysiology of epilepsy. This neurological disorder is characterized by a persistent predisposition of the brain to develop epileptic seizures, with a dysregulated neuroinflammatory response postulated to play a pathogenic role in this disease [152]. The expression profiles of IL-1R1 during seizures were found to be initially upregulated in the hippocampal neurons, followed by a delayed and transient expression in astrocytes in the limbic and extralimbic areas, suggesting that IL-1β may further enhance IL-1R1 production by acting in an autocrine manner or by paracrine signaling [153,154]. The IL-1R1/TLR4 signaling pathway has been widely studied due to its potential role in the initiation and exacerbation of seizures. Their endogenous ligands—IL-1β and HMGB1, respectively—were shown to be increased following acute seizure activities in murine models [155]. Importantly, Iori et al. found that blocking the IL-1R1/TLR4 pathway via caspase-1 inhibitor VX-765 and an investigational TLR4 antagonist effectively delayed disease progression and significantly reduced chronic seizures by 90% [156]. These observations are further supported by growing evidence showing that inhibiting IL-1R1 via genetic or pharmacological means results in a significant reduction in seizure susceptibility [157,158]. Recently, anakinra has been administrated to children with febrile-infection-related epilepsy syndrome (FIRES), effectively reducing seizure frequency [159,160].

### 4.7. IL-1R1 in Traumatic Brain Injury and Stroke

The role of IL-1β in neurodegeneration following ischemic brain injury has been the subject of a significant number of studies. The increase in cytokine levels occurs at an early stage (from 0 to 3 h postischemia), with the main source of IL-1β postulated to be microglia, and is sustained during later stages due to the delayed expression by astrocytes, neurons, endothelial cells and other immune cells [161,162]. On one hand, in animal models of cerebral ischemia, IL-1β has been shown to markedly increase cell damage and exacerbate injury [163,164]. On the other hand, a wealth of preclinical studies reported that blocking IL-1 signaling through the administration of IL-1Ra provided neuroprotective effects, with decreased tissue loss and attenuated cognitive deficits, in ischemic stroke [165,166,167]. Likewise, reduced neuronal damage has been observed after IL-1Ra exposure or treatment with anakinra in experimental traumatic brain injury (TBI) models [168,169,170]. Further experimental studies by Basu and colleagues using *IL-1R1* knockout mice revealed that the absence of IL-1 signaling after brain injury has a direct correlation with substantial reductions in microgliosis and astrogliosis as well as with IL-6 and COX-2 production, highlighting the importance of this receptor in microglial activation [47,55]. Together, these and similar observations prompted clinical trials of both IL-1Ra and anakinra for ischemic and hemorrhagic stroke, yielding significant reductions in the levels of inflammation and improvements in cognitive functions when given to patients in the early stages after a stroke [171,172,173].

### 4.8. IL-1R1 in Prion Diseases

Prion diseases, also known as transmissible spongiform encephalopathies, are a group of progressive and fatal neurodegenerative disorders characterized by deposition of an abnormal isoform of the host-encoded prion protein (PrP), astrogliosis and microgliosis, and neuronal loss. They may be sporadic (e.g., sporadic Creutzfeldt–Jakob disease), genetic (e.g., genetic Creutzfeldt–Jakob disease, Gerstmann–Sträussler–Scheinker syndrome, fatal familial insomnia) or infectious (e.g., kuru) in origin [174]. The involvement of IL-1 cytokines in prion pathogenesis has been assessed in several studies using murine models, revealing glial overexpression of pro-inflammatory cytokines IL-1α and IL-1β [175,176,177,178]. Nonetheless, one study was unable to detect secretion of IL-1β in mice with prion disease [179]. These asymmetric findings may be related to the diversity of the mouse models used for studying prion diseases or may reflect distinct disease stages. Mice lacking IL-1R1 were reported to show a significant delay in the accumulation of the misfolded isoform of the prion protein compared to wild-type controls [180,181]. According to the authors, the knockdown of *IL-1R1* may delay early-stage astrogliosis or enhance microglial phagocytosis of prions, indicating the IL-1/IL-1R1 signaling as an early driver of disease [182]. More recently, Bertani et al. showed that blocking IL-1R1 with anakinra normalized hippocampal neurotransmission and reduced seizure susceptibility in a transgenic mouse model of genetic Creutzfeldt–Jakob disease [74].

### 4.9. IL-1R1 in Other CNS Diseases

While the exact mechanisms by which IL-1R1 operates in the brain remain elusive, it is clear that the function of this protein extends beyond the CNS diseases highlighted on the above sub-sections. For instance, Todd et al. found out that IL-1β secreted by microglia and interacting with IL-1R1 expressed by astrocytes provide neuroprotection in excitotoxin-damaged mouse retinas [43]. The authors observed that treatment of retinas with IL-1β stimulated the activation and proliferation of microglia, resulting in increased neuronal survival following an excitotoxic insult. Interestingly, this neuroprotection was significantly reduced in mice lacking IL-1R1; however, it was rescued when expression of IL-1R1 was selectively restored in astrocytes, suggesting that astrocytic IL-1R1 is essential for the efficient activation of microglia and the induction of inflammatory responses in response to damage [43]. However, a more cytotoxic role for the IL-1β/IL-1R1 signaling complex in retinal degeneration has been proposed by others [183]. Recently, in a murine model of light-induced retinopathy study, it was observed that greater infiltration or activation of monocytes, macrophages and microglia was associated with increased levels of pro-inflammatory cytokines, including IL-1β, as well as photoreceptor cell death. The administration of anakinra and rytvela significantly downregulated: (i) the infiltration of monocytes, macrophages and microglia and maintained morphology of the cells in a ramified, steady-state; (ii) the pro-inflammatory mRNA levels *IL-1β, IL-6* and *CCL2;* (iii) the apoptosis of photoreceptors [184].

Meanwhile, a recent study showed that a lack of the interleukin 1 receptor 8 (IL-1R8) or excessive IL-1β signaling impacts the neuron synapse morphology, plasticity and function through the over-activation of IL-1R1. Both strategies led to the upregulation of the mechanistic target of rapamycin (mTOR) pathway and increased levels of the epigenetic regulator methyl-CpG-binding protein 2 (MeCP2), which is critically involved in neurological diseases characterized by defective plasticity, impaired cognition and intellectual disability. Interestingly, pharmacological treatment with anakinra restored MeCP2 expression and cognitive deficits in IL-1R8-deficient mice, highlighting that the inhibition of IL-1 signaling may be efficacious for treating neurological diseases through immune system modulation [4,185].

## 5. Tractability of IL-1R1 as a Pharmacological Target

As highlighted above, there is mounting evidence of a causal role of dysregulated activity of IL-1R1 signaling in multiple CNS diseases. Notwithstanding, to maximize the likelihood of success in modulating IL-1R1 signaling and further drug discovery endeavors, characterization in terms of tractability and druggability is of paramount importance. The constant advances in the fields of structural bioinformatics, virtual screening and fragment-based lead discovery, coupled with those also witnessed in the field of phenotypic screening, should encourage and guide the design and optimization of targeted therapies.

### 5.1. Availability of Protein Structures

**Figure 4:**
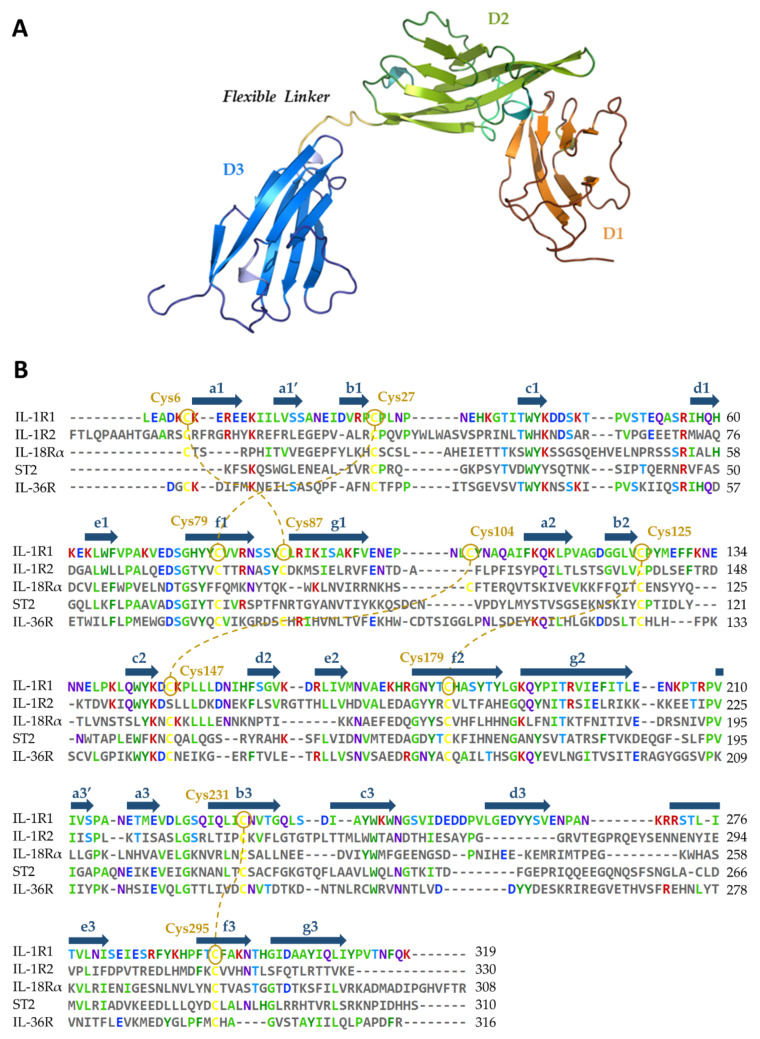
(**A**) Molecular structure the IL-1R1 ectodomain. The coordinates were retrieved from the PDB (PDB entry 4GAF). The Ig-like domains are labeled as D1 (orange), D2 (green) and D3 (blue), and the 6 amino acid flexible linker located between D2 and D3 is represented in yellow. (**B**) Alignment of IL-1R1, IL-1R2, IL-18Rα, ST2 and IL-36R ectodomains. The β-strands were assigned using the DSSP algorithm [191], and are shown for the IL-1R1 sequence (a1-g1 located in D1, a2-g2 located in D2 and a3-g3 located in D3, represented as blue arrows). Brown circles show the conserved cysteine residues, and the connectivity between the disulfide bonds in the IL-1R1 structure is represented by brown dashed lines. IL-1R1 shares 25.5%, 18.4%, 19.6% and 29.3% sequence identity with IL-1R2, IL-18Rα, ST2 and IL-36R, respectively. The alignments were generated using the Clustal Omega Server [192]. Adapted from [193]. Figure abbreviations: IL-1R1, interleukin-1 receptor type 1; IL-1R2, interleukin-1 receptor type 2; IL-18Rα, interleukin-18 receptor-α; ST2, defined as interleukin-33 receptor; IL-36R, interleukin-36 receptor.

Understanding the structural intricacies of IL-1R1 is pivotal not only to dissect general mechanisms of signal activation and inhibition via the IL-1 cytokines, but also to rationalize and predict feasible binding regions for the development of modulators. IL-1R1 is a transmembrane signaling receptor composed of a 319 amino acid ectodomain sitting on top of a question-mark-shaped structure supported by a transmembrane domain (TM) and an intracellular 217 amino acid TIR domain. Vigers et al. laid the groundwork for the structural elucidation of the IL-1R1 ectodomain (henceforth referred to as IL-1R1-ECD), describing the 3D crystal structure of this region determined at 2.5 Å resolution [186]. The IL-1R1-ECD folds into three Ig-like domains, which are referred to as D1, D2 and D3. Each domain is characterized by an extensive β-structure composed of seven to nine strands, arranged in a two-layer sandwich, which are stabilized by disulfide bonds involving pairs of highly conserved cysteine residues in the IL-1 receptor family (Figure 4). The two Ig-like domains, D1 and D2, are linked by a disulfide bond, separated from D3 via a 6 amino acid flexible linker lacking a secondary structure. As of December 2021, a total of 5 crystal structures of protein complexes containing the extracellular domain of IL-1R1 could be found in the Protein Data Bank (PDB) (Table 1) [187]. Of these, four receptor-bound structures adopt an open conformation, namely the PDB entries 1IRA [188], 1ITB [186], 4DEP [27] and 4GAF [189]. The remaining structure corresponds to a closed conformation of IL-1R1–PDB entry 1G0Y [190] (Supplementary Figure S1).

The flexibility of the linker between D2 and D3 is crucial for the positioning of D3 for cytokine binding, allowing the formation of a stable complex and then the recruitment of IL-1RAcP [27]. Structural alignments of the IL-1R1-ECD X-ray structures reveal structural differences in the D3 region, emphasizing the potential higher re-orientation ability of this domain when compared to the D1-D2 module. Interestingly, the structural complex of the IL-1R1-ECD with a small antagonist peptide is available (PDB entry 1G0Y), showing a 170° rotation of D3 relative to a reference D3 in the IL-1R1/IL-1β complex (PDB entry 1ITB), thereby exposing an unexpected binding mode for the peptide [190]. Recently, structural analyses employing a combination of small-angle X-ray scattering (SAXS) measurements with molecular dynamics (MD) simulations of the IL-1R1-ECD revealed an ensemble of closed conformations where D3 was also rotated, highlighting the role of this linker’s flexibility-dependent function in two structurally similar IL-1R1 ectodomains—IL-1RAcP-ECD and IL-18Rβ-ECD [194].

### 5.2. Relevant IL-1R1–Protein Interactions

**Figure 5:**
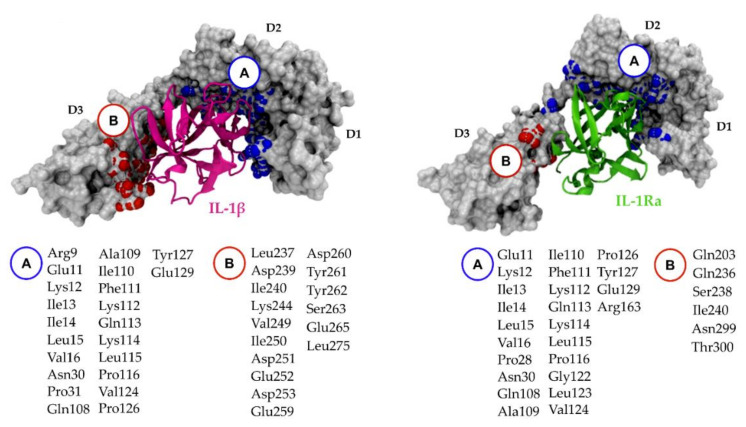
Structures of the binary IL-1R1-ECD/IL-1β (PDB entry 1ITB) and IL-1R1-ECD/IL-1Ra (PDB entry 1IRA) complexes. In both panels, IL-1R1-ECD is depicted in the surface representation (gray), while IL-1β (magenta) and IL-1Ra (lime) are shown in cartoon representations. The two binding regions are shown as spheres and colored in blue (site A) and red (site B), respectively. Left panel: IL-1β interacts with the two sites on the receptor surface (IL-1R1-ECD residues involved are presented in blue and red). Right panel: IL-1Ra interacts mostly with site A (IL-1R1-ECD residues involved are presented in blue and red). Figure abbreviations: IL-1β, interleukin-1β; IL-1R1-ECD, extracellular domain of IL-1R1; IL-1Ra, interleukin 1 receptor antagonist.

Fields et al. offered a remarkable analysis of the molecular mechanisms involving the IL-1 signaling complex formation [6]. Upon complex formation, the IL-1R1 structure essentially does not change, maintaining the core β-structure and domain fold. The IL-1R1-ECD adopts a question-mark-shaped architecture, forming two distinct binding regions, site A and B, which drive the interactions with IL-1 cytokines (Figure 5). Site A of the receptor is located in the Ig-like D1 and D2 interface, while the D3 region harbors site B. Site-directed mutagenesis of IL-1β revealed that three important residues, Arg11 [195], His30 [196] and Gln32 [186], anchor the cytokine onto the receptor surface. The former two residues establish interactions with domain D2 of IL-1R1-ECD (Phe111, Lys112, Gln113, Val124, Pro126 and Tyr127), while the latter binds the D1-D2 interface (Ile14, Leu15, Val16, Gln108, Ala109, Ile110 and Phe111). Upon IL-1β binding, the contacts established at site B are essential to induce a conformational change in domain D3 of IL-1R1, further recruiting IL-1RAcP to initiate signal transduction (PDB entry 4DEP). In contrast, most of the interactions of antagonist IL-1Ra with IL-1R1 (PDB entry 1IRA) are established with domains D1 and D2, exhibiting only a few contacts with D3 [188]. This binary complex is not able to recruit IL-1RAcP, pointing out that the D3 domain of IL-1R1-ECD is fundamental for binding IL-1RAcP, and is consequently required for signaling. Site-directed mutagenesis studies identified key residues at the IL-1Ra surface (Trp-16, Gln-20, Tyr-34, Gln-36 and Tyr-147) contributing to strong binding interactions with IL-1R1-ECD [197]. In a similar way to IL-1β, these residues establish contact mostly through a network of residues of the D1-D2 interface.

The core residues of the IL-1R1 antagonist peptide (referred to as AF10847), from the experimental X-ray closed conformation of the IL-1R1-ECD (PDB entry 1G0Y), establish interactions at the D1-D2 interface, stabilizing IL-1R1-ECD in a closed conformation. Within the core, Gln15 of AF10847 makes multiple hydrogen bonds with Val16 and Ala109, being essential for receptor binding [190]. Therefore, from a pharmacological point of view, the D1-D2 interface is a promising site for small-molecule design because disruption of this interface could prevent cytokine binding (IL-1α and IL-1β) or stabilize the ECD in a closed conformation. Nonetheless, targeting the D3 domain may also represent a viable strategy, as small molecules can potentially impact the relative orientation of D3 and further recruitment of IL-1RAcP [27]. Pockets near both interfaces appear to be promising candidate binding sites for the development of therapeutics targeting IL-1R1.

### 5.3. Putative IL-1R1 Druggable Binding Sites

**Figure 6:**
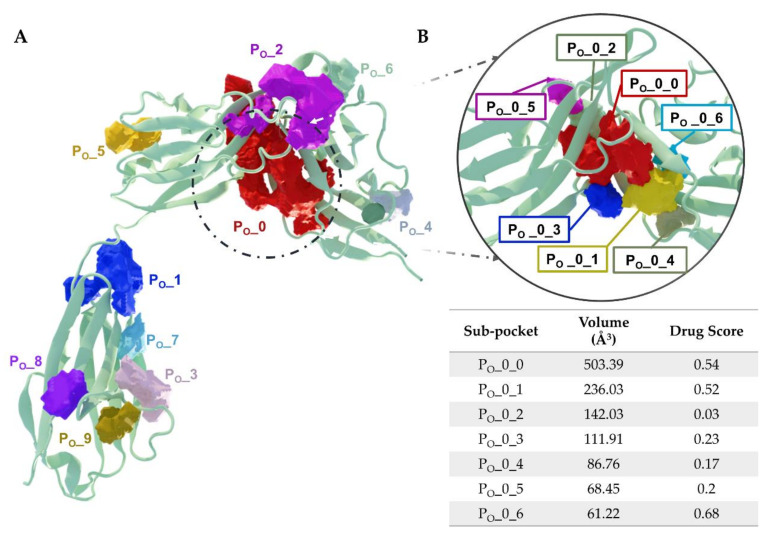
Binding pocket prediction on the IL-1R1 ectodomain surface (PDB entry 4GAF), using the DoGSiteScorer tool. (**A**) Ten binding pockets are shown: P_O__0 (red); P_O__1 (blue); P_O__2 (purple); P_O__3 (mauve); P_O__4 (dark grey); P_O__5 (yellow); P_O__6 (green); P_O__7 (cyan); P_O__8 (violet); P_O__9 (brown). (**B**) The sub-pockets comprising P_O__0 are illustrated in detail, with respective volume and druggability scores. Seven sub-pockets are shown: PO_0_0 (red); P_O__0_1 (yellow); P_O__0_2 (lime); P_O__0_3 (blue); P_O__0_4 (green); P_O__0_5 (purple); P_O__0_6 (cyan).

Yang provided the first hints for potential druggable binding sites on the IL-1R1-ECD surface [198], using the Schrödinger SiteMap algorithm [199]. Three different regions on the receptor surface were predicted to bind small molecules: (i) the D1-D2 interface region; (ii) the flexible linker between the Ig-like domains D2 and D3; (iii) the interface region between D1 and D3 when IL-1R1-ECD adopts a closed conformation. To add to the binding site analysis performed by Yang, here we present a binding site druggability analysis using the DoGSiteScorer algorithm [200]. DoGSiteScorer is a grid-based method and support vector machine (SVM) approach used to learn from 3D protein structures and detect binding pockets and sub-pockets holding high likelihood of attracting and accommodating drug-like ligands. A simple druggability score (with values varying between 0 and 1) is the output for each pocket and the respective sub-pockets. The higher the score, the higher the likelihood of the pocket being druggable. Our binding site analyses were performed on the crystallographic open conformation of IL-1R1-ECD at the best available resolution of 2.15 Å (PDB entry 4GAF). Table 2 presents the druggability scores and calculated descriptors for the major cavities identified at the IL-1R1-ECD surface (Figure 6A). According to the analysis, there are 10 identified potential binding pockets within the IL-1R1-ECD open conformation (pockets referred as P_O_) disclosing druggability scores ranging from 0.18 to 0.84. Three pockets P_O__0, P_O__1 and P_O__2 show druggability scores higher than 0.70, a large pocket volume, a high depth as well as an adequate hydrophobicity ratio, suggesting that there is a high chance of binding small molecules in these regions. In particular, the two predicted pockets P_O__0 and P_O__1 overlap with residues that are critical for ligand binding (as referred to in the previous section).

The decomposition of the largest predicted pocket (P_O__0) to its sub-pockets (Figure 6B) reveals three buried regions, P_O__0_0, P_O__0_1 and P_O__0_6, which are close to each other and have druggability scores of 0.54, 0.52 and 0.68, respectively. The residues lining these sub-pockets are as follows:

- P_O__0_0: Leu15, Val16, Ser17, Ser18, Glu21, Asp23, Val24, Arg25, Pro26, Ala69, Cys104, Tyr105, Asn106, Ala109, Phe111, Pro126, Tyr127, Met128, Glu129, Phe130, Phe131, His180, Ile192, Thr193, Arg194 and Val195;
- P_O__0_1: Glu11, Ile13, Ile14, Leu15, Arg25, Pro26, Pro28, Trp40, Ile92 and Tyr127;
- P_O__0_6: Arg25, Trp40, Leu64, Phe66, Tyr77, Ile92, Ser93, Ala94 and Phe96.

These buried sub-pockets present volumes that seem appropriate for ligand binding and are surrounded by other smaller pockets, suggesting that the D1-D2 interface may be suitable for targeting by small molecules and even for fragment-based approaches. Additionally, the presence of P_O__2, presenting the highest druggability score of 0.84, in the vicinity of P_O__0 is a good indicator for the D1-D2 interface as an appealing binding site for small-molecule therapeutics targeting this protein. The second top-ranked binding site P_O__1, with a volume of 575.29 Å^3^, a surface area of 1161.23 Å^2^ and a druggability score of 0.71, features residues of the flexible linker between D2 and D3, as well as D3 residues. From a pharmacological viewpoint, a druggable region located in close proximity to the flexible linker renders it a potential binding site for a small-molecule modulator. The flexible linker mediates conformational changes in the IL-1R1-ECD. As such, targeting this region may lead to a competitive small molecule trapping the receptor into an inactive conformation by hindering its required linker-mediated conformational transitions or by directly blocking the interaction of IL-1R1 with IL-1RAcP when binding only to D3. In summary, therapeutics targeting the D1-D2 interface or the D3 domain should be capable of interfering with cytokine binding or disrupting the more relevant and persistent protein–protein contacts, blocking the formation of the fully active heterotrimeric IL-1 signaling complex (IL-1β/IL-1R1/IL-1RAcP). Based on DogSiteScorer analyses disclosed here, together with the binding site analyses performed by others [198], it seems plausible that the highest scoring-predicted pockets represent promising focus points for targeting IL-1R1.

### 5.4. Ligand-Binding Site Differences across IL-1R1 Orthologs

**Figure 7:**
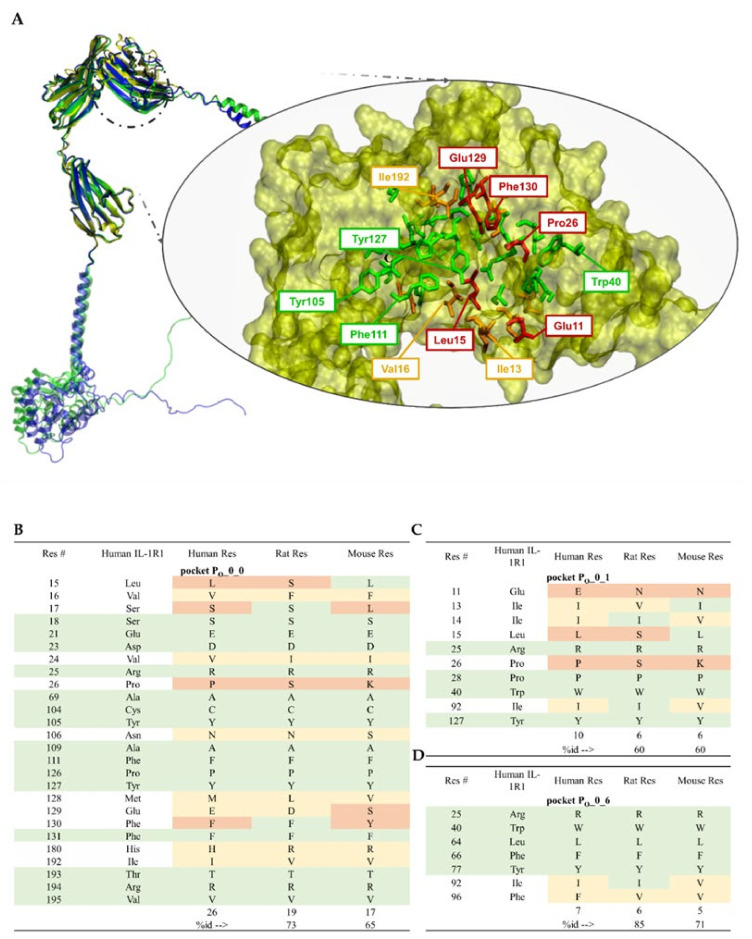
Comparison of human, rat and mouse interleukin-1 receptor type 1 (IL-1R1) protein sequences and structure. Upper panel: (**A**) Structural alignment of predicted rat (rIL-1R1, colored in green) and mouse (mIL-1R1, colored in blue) structures (full-length) retrieved from the AlphaFold Protein Structure Database [201] with the crystallographic structure of the extracellular domain of human IL-1R1 (hIL-1R1, colored in yellow) (PDB entry 4GAF). The protein structures were superimposed using the MatchMaker tool of the UCSF Chimera 1.15 software program [202]. The D1-D2 surface representation enclosed by the black circle is magnified, along with the conserved and non-conserved residues for the largest pocket (P_O__0) predicted by DogSiteScorer. Lower panel: Protein sequence alignment for residues (one-letter code) comprising three “druggable” binding pockets—P_O__0_0 (**B**), P_O__0_1 (**C**) and P_O__0_6 (**D**) predicted in the crystal structure of hIL-1R1 by DoGSiteScorer [200]. The sequence alignment was produced using the Clustal Omega Server [192]. Conservation across orthologs is color-coded as green, for full conservation across the three orthologs (a whole green table line represents 100% conservation); yellow represents amino acid differences with similar side-chain properties; red represents amino acid differences with distinct side-chain properties.

The information on whether a putative binding pocket on the target protein is structurally conserved may be relevant for the selection of an appropriate binding region for devising structure-based and virtual screening strategies. Differences in receptor structure and ligand-binding properties between ortholog species should also be considered when performing pharmacological testing experiments, both from efficacy and safety viewpoints. The availability of the gene sequences and molecular structures from different organisms represents an opportunity to analyze differences in the composition and conformation of ligand binding sites between orthologs. For comparison with the sequence and structure of human IL-1R1 (*Homo Sapiens*, UniProt code P14778), herein we used the IL-1R1 sequences of mice (*Mus Musculus*, Uniprot code P13504) and rats (*Rattus Norvegicus*, UniProt code Q02955), since both are the most common animals used in preclinical drug testing, and obtained their predicted molecular structures from the AlphaFold Protein Structure Database [201] (Figure 7A).

A comparison of the sequence and structure of the two large anchoring sites of IL-1β to the human IL-1R1 (hIL-1R1) and the rat IL-1R1 (rIL-1R1) reveals 8 conserved residues out of 19 sequence-matched residues lining receptor site A and 9 conserved residues out of 16 sequence-matched residues lining receptor site B (Supplementary Figure S2). These residues represent 17 out of 35 residues that are also conserved in mouse IL-1R1 (mIL-1R1), overall revealing sequence identity levels of 42% and 47% for site A in rats and mice, respectively, and identity levels of 56% and 50% for site B. We also compared three putative binding sites predicted in the crystallographic structure of IL-1R1-ECD (PDB entry 4GAF) by the DoGSiteScorer algorithm [200]. As anticipated, each of these regions individually are more conserved than the overall IL-1β binding sites (A and B).

Three smaller pockets can be detected within the largest pocket (P_O__0) and receive high druggability scores (Figure 7B). The overall sequence identity between hIL-1R1 and rIL-1R1 and mIL-1R1 is greater than 60%: (i) pocket P_O__0_0 presents 7 and 9 non-conserved residues out of 27, with a sequence identity of 73 and 65% against rIL-1R1 and mIL-1R1, respectively; (ii) pocket P_O__0_1 presents 4 non-conserved residues out of 10 residues compared to both rIL-1R1 and mIL-1R1 (sequence identity of 60%); (iii) the smallest of these three pockets, P_O__0_6, shows 1 and 2 out of 7 non-conserved residues (sequence identity of 85 and 71% for rIL-1R1 and mIL-1R1, respectively). Out of the three orthologs, mIL-1R1 is the only one that does not present a negatively charged side-chain, i.e., either aspartate (D) or glutamate (E), at position 129, which is known to be involved in the interaction with IL-1β or IL-1Rα. In addition, neither mIL-1R1 nor rIL-1R1 present a negatively charged residue at position 11 as in hIL-1R1, instead showing a glutamine (N). Within the core of the pocket, residue 26 is different across the three orthologs, with hIL-1R1 presenting a proline (P) and rIL-1R1 and mIL-1R1 showing a serine (S) and a positively charged lysine (K), respectively.

Focusing on the predicted binding pocket P_O__1, which is located in the D3 domain, this site presents sequence identity levels of 60 and 68% to hIL-1R1, respectively, compared to rat and mouse orthologs (Supplementary Figure S3). Significant differences between orthologs pertain to two residues of the 6 amino acid linker, human P206 and T207, which are mutated to arginine (R) and aspartic acid (D), respectively, in rIL-1R1 and mIL-1R1, as well as the presence of N301 in mIL-1R1 instead of H301 observed in hIL-1R1 and rIL-1R1. Human IL-1R1 shares 55% and 48% amino acid identity with their rat and mouse orthologs at binding site P_O__2, evidencing a lower degree of conservation between species in this region (Supplementary Figure S3). Overall, the two top-ranked binding sites, P_O__0 and P_O__1, exhibit relatively high sequence and structural conservation, which is consistent with their performing an important structural or biological role, as some of the residues lining these sites are crucial for IL-1 cytokine interactions.

## 6. Animal Disease Models Relating to IL-1 Pathways

Biomedical research involves the use of animal models to study different pathophysiologies and treatments. To be useful in preclinical research and recognized by regulatory authorities, such models must in principle be predictable, recapitulate human phenotype and pathology and produce results that can be extrapolated and transposed and that are often better than in vitro studies or computer models. Animal disease models (usually rodents) can also be of value in providing a safety assessment in the context of the molecular target, molecular mechanism or disease, and in some cases may demonstrate toxicological responses more representative of the response in patients. To develop animal models, numerous methods are used, including chemical exposure, genetic ‘knockout’ or ‘knock in’ or forward genetic modelling. Particularly valuable are transgenics expressing dominant oncogenes, siRNAs and those with telomerase inserts. Humanized transgenic animal models can be used for efficacy and toxicology studies to produce better read-outs, but they are difficult to obtain and expensive to maintain.

It has been shown that a robust neuroinflammatory response, with significantly elevated levels of IL-1β, is generally provoked within minutes in animals with induced traumatic brain injury (TBI). The presence of several soluble markers of neuroinflammation was evident in the region of brain trauma but also in CSF, as typified by pro-inflammatory cytokines and chemokines. Under pathological conditions in the CNS, chemokines are generated by activated resident local astrocytes and microglia and act to recruit peripheral immune cells into the brain parenchyma. In addition to chemokines, various cytokines are also generated and secreted by activated glia following TBI. For example, several studies in mice and rats aimed to characterize and quantify the biochemical changes and expression of inflammatory mediators after brain injury [203,204]. Following a controlled cortical impact (CCI) on young adult male rats, the levels of chemokines CXCL1, IFN-γ and TNF-α significantly peaked at 4 h postinjury compared to levels found in naïve or contralateral tissue. CXCL1, IFN-γ and TNF-α levels were then observed to decrease by at least 3-fold by 12 h postinjury. IL-1β, IL-4 and IL-13 levels were also significantly elevated at 4 h postinjury, although their expression did not decrease more than 3-fold for up to 24 h postinjury. Moreover, IL-1β and IL-4 levels displayed a biphasic temporal profile in response to injury, suggesting a role in adaptive immune responses [205].

As alluded in the introductory section, IL-1β, which regulates acute and chronic neuroinflammatory responses, is elevated in AD patients. In 2007, O’Banion and co-workers reported an inducible IL-1β overexpression model of chronic neuroinflammation—the IL-1β excisional activation transgenic (XAT) mouse model [206]. In this model, prolonged IL-1β elevation induces microgliosis and astrogliosis accompanied by a chronic increase in the pro-inflammatory cytokines IL-6 and TNF-α. When the inducible transgene is activated, neuroinflammation can last up to 10 months. IL-1β transgenic mice showed a dualistic histopathological appearance with respect to the characteristics of AD. On the one hand, the production and processing of the amyloid precursor protein (APP) was unchanged despite prolonged overexpression of the transgene, and the amyloid load was even reduced in hybrid IL-1β XAT and APP/PS1 Tg mice [206,207]. On the other hand, in IL-1β XAT and 3xTg AD mice this resulted in significant exacerbation of tau hyperphosphorylation within 1 month after IL-1β overexpression [208]. It was hypothesized that in this model, IL-1β induces the activation of microglia without placing it in a dysfunctional hyperreactive state, thereby sparing its ability to reactivate the phagocytic mode in favor of the clearance of Aβ aggregates [209]. It is also noteworthy that the IL-1β XAT mouse model showed no apparent neuronal loss or apoptosis within 2 and 5 months of IL-1β overexpression, respectively [206,207]. However, the animals manifested significant cognitive deficits, including contextual fear memory and spatial memory defects within 3 months of transgene induction [210].

The different effects of IL-1β overexpression on Aβ and tau pathology can be explained within the context of the inflammatory hypothesis of AD by Krstic and Knuesel [211]. Based on this hypothesis, tau hyperphosphorylation is an early neural response to neuroinflammatory stress, while Aβ pathology occurs after a shift of microglia to a more pro-inflammatory phenotype—as opposed to homeostatic (anti-inflammatory) conditions. This implies that microglial cells retain their physiological function in the IL-1β XAT mouse model [212] in contrast to immune-challenged animal models (for example, LPS and PolyI:C injection) [213,214,215] or neurotoxin (i.e., STZ) models [216].

Overall, the IL-1β model failed to reproduce the main lesions of the AD pathology despite the confirmation of the association between chronic neuroinflammation and cognitive deficits. This could mean that the model may not be suitable for studying sporadic AD. Nevertheless, while the pipeline of therapeutics targeting IL-1 pathways continues to move forward into the clinic and proof of concept IL-1 mechanisms, such models may still hold a place in the study of novel experimental therapies.

IL-1RAcP is of paramount importance for the regulation of not only IL-1α and IL-1β, but also of IL-33 and IL-36 cytokines (IL-36α, IL-36β, IL-36γ). IL-1RAcP forms functional receptor complexes with IL-1R1, IL-33R (ST2) and IL-36R, initiating different inflammatory pathways [6]. Recently, two late-onset Alzheimer’s disease mouse models with knock-out alleles of the *IL-1RAcP* were made available by the Jackson Laboratory. One of these models is a triple mutant line carrying a humanized APOE4 allele and the p.R47H mutation knocked into mouse Trem2 (besides the knock-out allele of the *IL-1RAcP* gene); the other model also preserves humanized Aβ and APOE4 knock-ins and CRISPR/Cas9-generated APP allele, p.R47H point mutation of the Trem2 gene and deletion of exon 3 of the IL-1RAcP—all intended to increase the risk of late-onset Alzheimer’s disease. Given the critical role of IL-1RAcP as a mediator of the inflammatory landscape, these mouse models may shed light on new mechanisms of neuroinflammation involving the IL-1 pathways and foster the development of therapeutics targeting the accessory protein.

## 7. Pipeline of IL-1 Therapeutic Modulators

The excessive stimulation of the IL-1/IL-1R1 molecular networks is associated with the pathogenesis of several disorders, extending from autoinflammatory diseases such as familial Mediterranean fever (FMF) and deficiency in IL-1 receptor antagonist (DIRA), to rheumatoid arthritis, type 2 diabetes, cancer and neuroinflammation-associated neurodegenerative diseases. Consequently, IL-1 has drawn considerable attention as a potential therapeutic target. Numerous avenues for blocking interleukin-1 in this broad spectrum of diseases have been exploited by the pharmaceutical industry and academics in the last decades (Table 3).

Several reviews can be found in the literature describing in great detail potential pharmacotherapies developed to modulate IL-1 pathways [6,10]. For the sake of concision, the next paragraphs focus only on therapeutics directly targeting IL-1R1.

### 7.1. Development Status of Therapeutics Targeting IL-1R1

**Figure 8:**
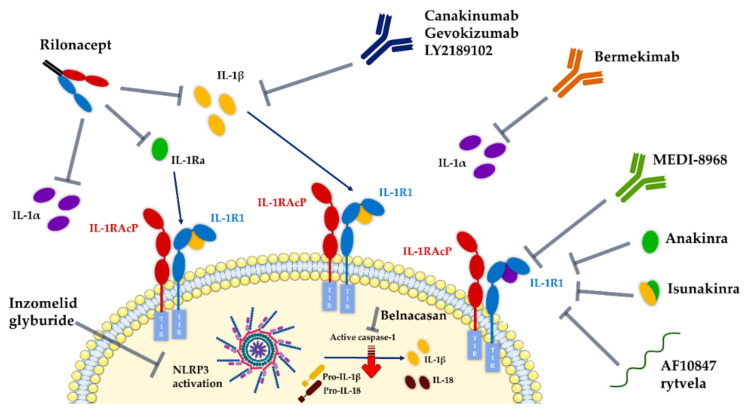
Main strategies targeting IL-1 signaling. Therapeutic approaches targeting IL-1R1 include a recombinant form of IL-1Ra (anakinra), a chimeric IL-1Ra-IL-1β protein (isunakinra), a fully human monoclonal antibody (MEDI-896) and peptides AF10847 and rytvela. Rilonacept stands as an engineered dimeric fusion protein consisting of the ECDs of the human IL-1R1 and IL-1RAcP linked to the Fc portion of human immunoglobulin G1 (IgG1), acting as a decoy receptor, binding IL-1α, IL-1β and IL-1Ra. The monoclonal antibodies (mAb) antagonizing IL-1β are canakinumab, gevokizumab and LYS2189102, whilst bermekimab works by blocking IL-1α. The small molecules inzomelid and glyburide directly inhibit NLRP3 inflammasome activation, while belnacasan specifically inhibits caspase-1 and consequently the maturation of pro-IL-1β into the respective active form. The figure was prepared using BioRender. Adapted from [243]. Figure abbreviations: IL-1, interleukin-1; IL-1R1, interleukin-1 receptor type 1; IL-1Ra, interleukin 1 receptor antagonist; IL-1RAcp, interleukin-1 receptor accessory protein; TIR domain, the toll-interleukin-1 receptor homology domain; NLRP3, NLR family pyrin-domain-containing 3.

Efforts to specifically target IL-1R1 have been focusing on antibodies or chimeric cytokine biologics (Figure 8):

- Anakinra (Kineret®, Amgen), a recombinant non-glycosylated form of the natural human IL-1Ra, distinguishable by the addition of an N-terminal methionine residue, reached the US and Europe drug markets in 2001 and 2002, respectively, to treat rheumatoid arthritis. Fundamentally, this molecule targets the IL-1R1-ECD, competitively inhibiting IL-1α and IL-1β binding and blocking intracellular signal transduction. Anakinra is rapidly removed from the body by renal filtration due to its small size (17.3 kD). However, it requires daily self-administration via subcutaneous injection, which may result in adverse side effects such as injection site reactions, missed doses and ultimately decreased patient treatment compliance [217,240]. Interestingly, a recent study reported that anakinra crossed a human in vitro model of the BBB derived from human umbilical cord blood stem cells at a 4–7-fold higher rate than the monoclonal antibodies bermekimab (IL-1α antagonist) and canakinumab (IL-1β antagonist) [241];
- EBI-005 (isunakinra), a human IL-1β and IL-1Ra chimeric protein, developed in 2013 by Eleven Biotherapeutics, has been shown to bind IL-1R1 at a higher affinity than IL-1β (KD = 0.014 nM for EBI-005; KD = 2.0 nM for IL-1β). This biologic was optimized for topical ocular administration in patients with dry eye disease and allergic conjunctivitis [189]. However, phase III clinical trials were halted after EBI-005 failed to achieve primary endpoints;
- The monoclonal antibody (mAb) AMG108 (licensed to AstraZeneca and Medlmmune and now termed MEDI-78998) binds IL-1R1-ECD, blocking IL-1β-mediated signaling pathways. In preclinical studies, this human mAb has shown efficacy in models of osteoarthritis; however, no significant clinical benefits were observed in phase II trials [242].

While biologics targeting IL-1R1 work relatively well at modulating IL-1 activity, an early goal of the field was to discover agonist or antagonist peptides with potential pharmaceutical application. In fact, it was back in 1996 that Yanofsky et al., by screening peptide phage display libraries, identified numerous small peptides binding to human IL-1R1-ECD, inhibiting IL-1-mediated cellular responses. By comparing their activity with IL-1α and IL-1β, they identified three different peptides exhibiting an IC_50_ below 3 nM [244]. One such peptide, AF10847 (21 amino acids), was later crystalized with IL-1R1 by Vigers et al., revealing an unexpected induced conformational change in the receptor [190]. Importantly, these data demonstrated the possibility of different orientations between structural domains (D1, D2, D3) of the extracellular domain of IL-1R1 and shed light on the ability of IL-1R1 to adopt multiple conformations that can be exploited by small-molecule modulators. Almost a decade later, a selective D-peptide antagonist of IL-1R1, termed rytvela, was developed based on the flexible portions of the IL-1RAcP, showing efficacy and potency in a broad variety of in vitro IL-1β-dependent assays [245], and more recently in reducing inflammation in models of neuro-retinal degeneration [184]. Approximately at the same time, a short peptide (ilantide) derived from the IL-1Ra, capable of binding to the IL-1R1-ECD, was shown to be able to reduce the inflammation state in the CNS and pancreatic islets [246].

### 7.2. New and Alternative Approaches Targeting IL-1/IL-1R1 Pathways

Although most IL-1R1-targeting therapeutics are antibodies and peptides, both have major limitations, including generally large molecular weights, difficult dosing frequencies, a lack of simple production processes and inconsistent or unfavorable pharmacokinetic (ADME) profiles. Small-molecule therapeutics may represent a more versatile option, combining adequate pharmacological properties (lower molecular weight, small polar surface area and lower hydrophilicity) and even potential allosteric modulation, which may in turn open avenues for the development of alternative solutions for targeting IL-1R1. Within the setting of compartmentalized CNS inflammation, an important aspect to take into consideration during the screening or design of IL-1R1 modulators is their ability to permeate barriers to the brain, such as the blood–brain barrier (BBB) and blood–cerebrospinal fluid barrier (BCSFB). In this way, the intersection between selective small molecules targeting brain IL-1R1 and the physicochemical properties required for CNS penetration is critical for development of efficacious strategies to modulate IL-1R1-mediated neuroinflammation [247,248,249]. Preliminary efforts to identify promising molecular scaffolds against IL-1R1 have been conducted in recent years [198,250,251]. However, to date, very few small molecules with promising in vivo efficacy to modulate IL-1R1-associated activities have been developed.

RNA interference (RNAi) technologies also represent promise for developing therapeutics targeting IL-1R1 signaling. One approach used short-hairpin RNA (shRNA) interference targeting IL-1R1 in the paraventricular nucleus (PVN), leading to reduced local IL-1R1 expression and downregulated sympathetic activity and improved myocardial remodeling, two important mechanisms underlying hypertension [252]. Silencing of *IL-1R1* gene expression through small-interference RNA (siRNA) in CD4+ T cells derived from relapsing-remitting multiple sclerosis patients inhibited in vitro Th17 cell differentiation via inhibition of interferon regulatory factor 4 (IRF4) expression. The authors highlighted that the therapeutic targeting of IL-1R1 may hold promise in Th17-related autoimmune disorders [119]. Using an adenoviral shRNA targeting IL-1β in rat models of spinal cord injury, Lin et al. showed that IL-1β downregulation significantly decreased the expression of pro-inflammatory TNF-α and pro-apoptotic Bax protein, whereas the levels of anti-apoptotic B-cell lymphoma-2 (BCL-2) were increased [253]. Importantly, this study reported that reduced IL-1β expression may promote neuronal survival and attenuate the severity of spinal cord injury. Similarly, knockdown of *IL-1β* by siRNA in hypoxic–ischemic brain injury in neonatal rat models attenuated neurologic deficit, brain edema and astrocyte swelling caused by hypoxia ischemia [254]. Taken together, these studies suggest that the downregulation of the IL-1R1/IL-1β signaling pathway by RNA interference may be neuroprotective in CNS disorders. The application of shRNA or siRNA molecules directed against IL-1R2 [255], IL-RAcP [256], NF-κB [257,258] or interleukin-1-receptor-associated kinase 1 (IRAK1) [259] have also been attempted. Clinical studies are required to assess their benefit in treating IL-1-associated human CNS diseases.

Small molecules directly interacting with IL-1R1 to modulate its activity or siRNA or shRNA specifically modulating *IL-1R1* gene expression can shed light on new therapeutic avenues against IL-1R1 and related pro-inflammatory mediators. From a pharmacology viewpoint, it is expected that along with the progress in elucidating IL-1R1 mechanisms in CNS and other diseases together with an increasing wave of structural knowledge, more IL-1R1-targeting agents will be available for clinical trials in the coming years.

## 8. Conclusions

IL-1, IL-1R1 and its downstream mediators play pivotal roles in the modulation of inflammatory responses against immune challenges. At the same time, IL-1 has also been shown to be involved in a wide range of human pathologies having a high prevalence and posing a striking socioeconomic burden. This double-edged sword behavior raises one important question: Is IL-1 a harmful driver of disease, a protective response or something in between? We believe it may be all the above, and subject to dependency on cell subtypes, the signaling sub-pathways involved and the stage of disease progression.

Despite promising in vitro and in vivo results in models of CNS diseases, the complexity of translating findings from animal studies to humans through feasible clinical trials, along with some conflicting data obtained in human studies, seem to have reduced the interest of pharmaceutical companies in IL-1R1 drug discovery. Nevertheless, the studies reviewed here make a strong case for IL-1R1 as a key player in CNS pathological conditions. Under these conditions, IL-1R1 is strongly activated by IL-1 secreted by surrounding glial cells, and the resulting inflammatory signal may lead to CNS tissue damage. An increasing impetus for exploring IL-1R1 as a therapeutic target has resulted from mounting non-clinical and clinical evidence on the use of IL-1R1-antagonizing biological therapeutics (biologics such as anakinra) showing encouraging correlations with substantial reductions in disease-linked microgliosis and astrogliosis, as well as downregulated levels of pro-inflammatory players such as IL-6 and COX-2.

As the field develops, the fine modulation of IL-1R1 activity may entail the definition of a novel paradigm combining small molecules or other therapeutic approaches such as antibodies or IL-1 suppression, for example through RNA interference. In this quest, the development of appropriate in vitro and in vivo disease models is of the utmost importance in determining the outcomes of modulating the IL-1 signaling pathway in the CNS. Given its direct role in modulating IL-1 overexpression and a myriad of other pro-inflammatory mediators involved in the neuroinflammatory landscape, as well as in sustaining inflammation in chronic neurodegeneration, targeting IL-1R1 in specific tissues or organs, particularly the CNS, may still reveal itself as a valuable therapeutic strategy against exacerbated neuroinflammatory processes linked to disease states of the CNS.

## Figures and Tables

**Table 1:** X-ray crystallographic structures of IL-1R1 deposited in the Protein Data Bank (as of December 2021).

PDB Entry	Resolution (Å)	R-Value (Work)	Pub Year	Description
1G0Y	3.00	0.223	2000	IL-1R1 complexed with antagonist peptide AF10847
1IRA	2.70	0.213	1997	IL-1R1 complexed with IL-1Ra
1ITB	2.50	0.229	1997	IL-1R1 complexed with IL-1β
4DEP2 chains	3.10	0.210	2012	IL-1R1 complexed with IL-1β and IL-1RAcP
4GAF	2.15	0.215	2013	IL-1R1 complexed EBI-005, a chimera of human IL-1β and IL-1Ra

Table abbreviations: Interleukin receptor type-1 (IL-1R1); interleukin-1β (IL-1β); interleukin-1 receptor antagonist (IL-1Ra); interleukin-1 receptor accessory protein (IL-1RAcP).

**Table 2:** The five largest pockets predicted by DoGSiteScorer on the IL-1R1 ectodomain surface (PDB entry 4GAF).

Pocket	Volume (Å^3^)	Surface (Å^2^)	Depth (Å)	Nº Residues	Hydrophobicity Ratio	HBA/HBD	Drug Score
P_O__0	1209.78	1641.79	26.31	55	0.37	86/28	0.82
P_O__1	575.29	1161.23	15.32	25	0.36	48/27	0.71
P_O__2	490.77	605.27	22.21	27	0.38	29/16	0.84
P_O__3	191.44	398.74	11.78	10	0.51	18/4	0.43
P_O__4	179.47	464.04	9.93	9	0.42	20/11	0.29

Table abbreviations: Hydrogen bond acceptors (HBA); hydrogen bond donors (HBD).

**Table 3:** Summary of therapeutic agents available for the modulation of IL-1 activity.

Therapeutic Agent	Type	Target	Company	Therapeutic Indication
**Anakinra**	Recombinant form of human IL-1Ra	IL-1R1	Amgen (now Swedish Orphan Biovitrum)	Rheumatoid arthritis ^1^ [217] Gout [218] CAPS ^1^ [219] Type 2 Diabetes [220] Cardiovascular disease [221,222] Giant Cell Arthritis (NCT02902731) ^2^ Subarachnoid Hemorrhage (NCT03249207) ^2^ Intracerebral Hemorrhage (NCT03737344, NCT04834388) ^2^ Multiple Sclerosis (NCT04025554) ^2^
**Rilonacept**	Fusion protein of IL-1RAcP, IL-1RI and IgG-Fc	IL-1β IL-1α IL-1Ra	Regeneron	Recurrent Pericarditis ^1^ [223] CAPS ^1^ [224] Gout [225]
**Canakinumab**	Human IgG1 monoclonal antibody	IL-1β	Novartis	CAPS ^1^ [226] TRAPS ^1^ [227] HIDS/MKD ^1^ [228] FMF ^1^ [229] Type 2 Diabetes [230] Still’s disease ^1^ [231] AD (NCT04795466) ^2^
**Gevokizumab**	Human IgG2 monoclonal antibody	IL-1β	Xoma	Behcet’s Uveitis [232]
**LY2189102**	Human IgG1 monoclonal antibody	IL-1β	Lilly	Type 2 diabetes [233] Rheumatoid arthritis (NCT00380744) ^2^
**Bermekimab (MABp1)**	Human IgG1 monoclonal antibody	IL-1α	XBiotech	Atopic Dermatitis [234]
**MEDI-8968 (AMG108)**	Human IgG2 monoclonal antibody	IL-1R1	MedImmune	COPD [235]
**Isunakinra (EBI-005)**	Human IL-1β and IL-1Ra chimeric protein	IL-1R1	Eleven Biotherapeutics	Dry eye disease [236] Solid Tumors (NCT04121442) ^2^
**AF10847**	peptide	IL-1R1	Array BioPharma	--
***rytvela***	peptide	IL-1R1	Elim Biopharmaceuticals	--
**Inzomelid**	Small molecule	NLRP3 inflammasome	Roche (previously Inflazome)	CAPS [237]
**Belnacasan (VX-765)**	Small molecule	Caspase-1	Vertex Pharmaceuticals	Rheumatoid arthritis [238] Epilepsy [239]

^1^ FDA approval for therapeutic indication; ^2^ clinical trial details can be accessed at www.clinicaltrials.gov (accessed on 23 December 2021). Table abbreviations: interleukin-1 receptor type 1 (IL-1R1); interleukin-1α (IL-1α), interleukin-1β (IL-1β); interleukin-1 receptor antagonist (IL-1Ra); NLR family pyrin domain containing 3 (NLRP3); Alzheimer’s disease (AD); Cryopyrin-associated periodic syndrome (CAPS); tumor necrosis factor receptor-associated periodic syndrome (TRAPS); hyperimmunoglobulin D syndrome/mevalonate kinase deficiency (HIDS/MKD); chronic obstructive pulmonary disease (COPD).

## Supplementary Materials

Supplementary information can be found at https://www.mdpi.com/article/10.3390/ijms23031731/s1.
