# Supplementary material for: The Therapeutic Prospects of Targeting IL-1R1 for the Modulation of Neuroinflammation in Central Nervous System Disorders
Source: Int J Mol Sci. 2022 Feb 2;23(3):1731. doi: 10.3390/ijms23031731 (PMC8915186; doi:10.3390/ijms23031731)
Supplement: Supplementary file 1 [file ijms-23-01731-s001.zip › ijms-1545562-supplementary.pdf]

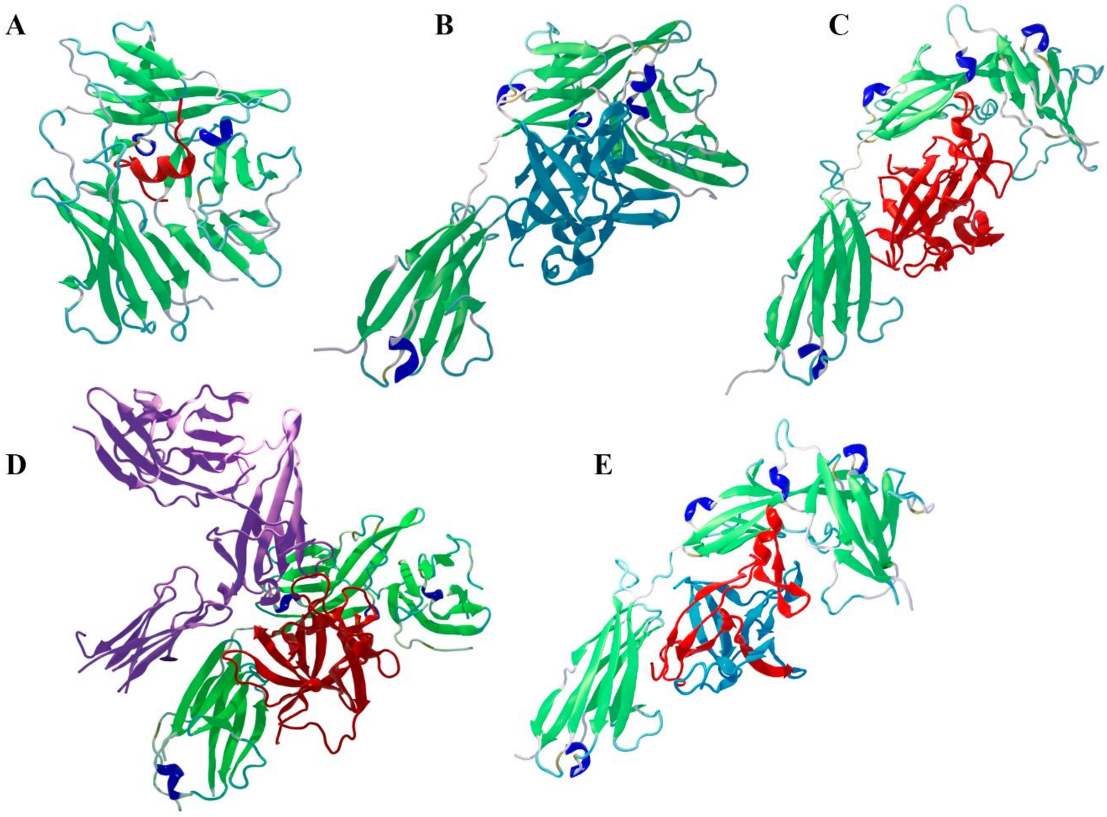

Figure S1. Three-dimensional (3D) representations of the extracellular domain of interleukin-1 receptor type 1 (IL-1R1-ECD)-ligand bound complexes available in the PDB. X-ray structure of IL-1R1-ECD bound (**A**) to a small antagonist peptide AF10847 (coloured in red), adopting a closed conformation (PDB entry 1G0Y); (**B**) to interleukin-1 receptor antagonist (IL-1Ra, coloured in blue) (PDB entry 1IRA); (**C**) to interleukin-1β (IL-1β, coloured in red) (PDB entry 1ITB); (**D**) to IL-1β (coloured in red) and the receptor accessory protein (IL-1RAcP, coloured in purple) (PDB entry 4DEP); and (**E**) to EBI-005, a chimera of human IL-1β and IL-1Ra (coloured in blue and red) (PDB entry 4GAF).

A

Res #	Human IL-1R1	Human Res	Rat Res	Mouse Res
		**Site A – IL-1β**		
9	Arg	R	Y	Y
12	Lys	K	E	Q
13	Ile	I	V	I
14	Ile	I	I	V
15	Leu	L	S	L
16	Val	V	F	F
30	Asn	N	T	T
31	Pro	P	P	P
108	Gln	Q	Q	Q
109	Ala	A	A	A
110	Ile	I	S	T
111	Phe	F	F	F
112	Lys	K	I	P
113	Gln	Q	Q	Q
114	Lys	K	R	R
115	Leu	L	L	L
116	Pro	P	H	H
127	Tyr	Y	Y	Y
129	Glu	E	D	S
		19	8	9
		%id -->	42	47

B

Res #	Human IL-1R1	Human Res	Rat Res	Mouse Res
		**Site B – IL-1β**		
237	Leu	L	F	F
239	Asp	D	D	D
240	Ile	I	L	L
244	Lys	K	K	K
249	Val	V	E	E
250	Ile	I	I	I
251	Asp	D	E	E
252	Glu	E	W	W
253	Asp	D	D	N
259	Glu	E	E	E
260	Asp	D	D	D
261	Tyr	Y	Y	Y
262	Tyr	Y	Q	Q
263	Ser	S	F	F
265	Glu	E	E	E
275	Leu	L	L	L
		16	9	8
		%id -->	56	50

Figure S2. Comparison of human, rat and mouse interleukin-1 receptor type 1 (IL-1R1) protein sequences and structure. Protein sequence alignment for residues (one-letter code) comprising (**A**) site A and (**B**) site B of IL-1R1, which establish interactions with IL-1β. The sequence alignment was produced using the Clustal Omega Server [192]. Conservation across orthologs is color coded as green, for full conservation across the three orthologs (a whole green table line represents 100% conservation); yellow, for amino acid differences with similar side-chain properties; red, for amino acid differences with distinct side-chain properties.

A

Res #	Human IL-1R1	Human Res	Rat Res	Mouse Res
		**Pocket P_{O}_1**		
202	Glu	E	D	D
203	Glu	E	D	E
204	Asn	N	S	N
205	Lys	K	K	K
206	Pro	P	R	R
207	Thr	T	D	D
208	Arg	R	R	R
209	Pro	P	P	P
210	Val	V	V	V
234	Thr	T	T	T
235	Gly	G	G	G
236	Gln	Q	Q	Q
237	Leu	L	F	F
238	Ser	S	T	S
239	Asp	D	D	D
240	Ile	I	L	L
265	Glu	E	E	E
270	Lys	K	K	K
271	Arg	R	R	R
272	Arg	R	K	K
273	Ser	S	Y	Y
298	Lys	K	K	K
299	Asn	N	N	N
300	Thr	T	T	T
301	His	H	H	N
		25	15	17
		%id -->	60	68

B

Res #	Human Res	Human 1L	Rat Res	Mouse Res
		**Pocket P_{O}_2**		
53	Gln	Q	K	R
60	His	H	Q	Q
61	Lys	K	N	N
63	Lys	K	H	H
65	Trp	W	W	W
125	Cys	C	C	C
128	Met	M	L	V
129	Glu	E	D	S
130	Phe	F	F	Y
131	Phe	F	F	F
132	Lys	K	K	K
133	Asn	N	D	D
134	Glu	E	E	E
138	Leu	L	L	L
139	Pro	P	P	P
141	Leu	L	V	V
179	Cys	C	C	C
180	His	H	R	R
181	Ala	A	T	M
182	Ser	S	S	S
183	Tyr	Y	Y	Y
184	Thr	T	T	T
185	Tyr	Y	Y	F
186	Leu	L	Q	R
192	Ile	I	V	V
193	Thr	T	T	T
194	Arg	R	R	R
		27	15	13
		%id -->	55	48

Figure S3. Comparison of human, rat and mouse interleukin-1 receptor type 1 (IL-1R1) protein sequences and structure. Protein sequence alignment for residues (one-letter code) comprising two binding pockets – (**A**) Po_1 and (**B**) Po_2 – predicted in the crystal structure of human IL-1R1 (hIL-1R1) by DoGSiteScorer [200]. The sequence alignment was produced using the Clustal Omega Server [192]. Conservation across orthologs is color coded as green, for full conservation across the three orthologs (a whole green table line represents 100% conservation); yellow, for amino acid differences with similar side-chain properties; red, for amino acid differences with distinct side-chain properties.
